# P-930. Infective Endocarditis and the Role of Oral Pathogens

**DOI:** 10.1093/ofid/ofae631.1121

**Published:** 2025-01-29

**Authors:** Tanna J Lauriente, Benjamin Leis, Satchan Takaya, Chantelle Y G Wong, Cara Spence, Mohan Teekasingh

**Affiliations:** University of Saskatchewan, Saskatoon, Saskatchewan, Canada; University of Saskatchewan, Saskatoon, Saskatchewan, Canada; University of Saskatchewan, Saskatoon, Saskatchewan, Canada; University of Saskatchewan, Saskatoon, Saskatchewan, Canada; University of Saskatchewan, Saskatoon, Saskatchewan, Canada; University of Saskatchewan, Saskatoon, Saskatchewan, Canada

## Abstract

**Background:**

Studies suggest poor oral health increases the risk of infective endocarditis (IE) as 20-53% of cases stem from organisms linked with oral flora. Despite intravenous drug use being a known risk factor for IE, our local data reveals nearly 33% of IE pathogens in individuals who inject drugs (PWID) come from oral microflora. Shockingly, only 9.1% of IE patients received dental consultation during admission.

**Table 1.**
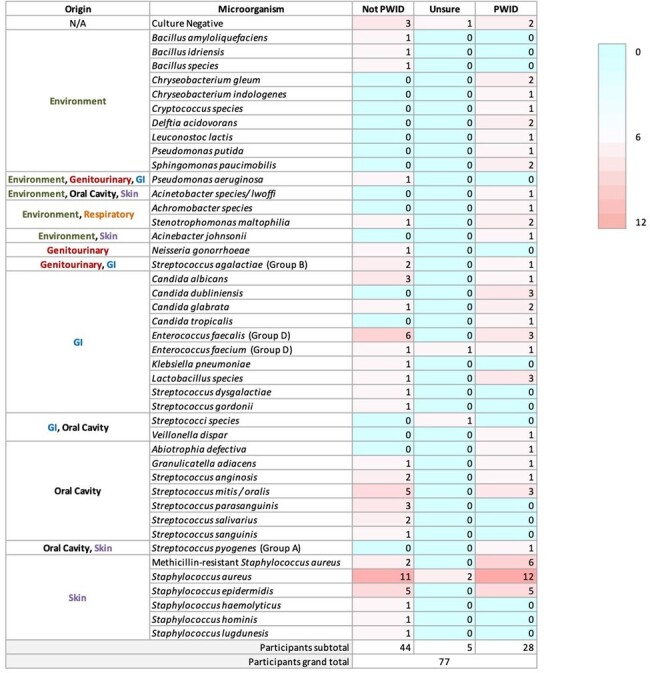
Classification of isolated infective endocarditis pathogens by site of origin and injection drug use status. 77 participants underwent retrospective chart review with 36.4% identifying as people who inject drugs (PWID). A heat map was used with blue indicating 0 and red indicating higher number of pathogens up to 12. This demonstrates that the majority of pathogens identified from positive blood cultures were from skin, oral cavity, and GI sources. However, among the PWID group, environmental pathogens were more prevalent.

**Methods:**

This is a pre-and-post intervention cohort study. Through retrospective chart review, we established baseline PWID status, pathogen identified in positive blood cultures, dentistry details, and determined origin site of the microorganism. We reviewed all adult IE admissions to our tertiary care hospital in Saskatoon, SK from January 1, 2022 to May 31, 2023 who met the Modified Duke Criteria. Using this data, we designed a Multi-Disciplinary Endocarditis (MENDO) Clinical Pathway to address gaps in dentistry review and intervention at our hospital. This intervention, which began September 25, 2023, has enrolled 35 patients so far.

**Results:**

Among the 77 retrospective participants meeting inclusion criteria, 36.4% were PWID and 31.2% of all IE pathogens were from oral sources. Commonly isolated oral pathogens included Viridans Group *Streptococci* and *Streptococcus anginosis*. Notably, 32.1% of IE organisms in PWID were oral pathogens which was similar to non-PWID (p = 1.0). In the retrospective cohort, the baseline rates of dentistry consultation and dental intervention were 9.1% and 7.8% respectively. Since September 2023, 35 patients have been enrolled in the MENDO pathway, 72.1% of which are PWID. Through care standardization and case worker support, our dentistry consultation rate and intervention rates are now 35.5% (p = 0.0115) and 9.7% (p = 0.714) respectively.

**Conclusion:**

In our study, approximately 1 in 3 PWID had IE due to oral pathogens. This was comparable to non-PWID, suggesting equal prevalence of IE due to dental sources in both populations. Our intervention enhanced dentistry access for PWID, and we hypothesize that our longitudinal data will show reduced infection relapse. Integrating oral health assessments and interventions into the care of individuals at risk for IE, including those with PWID, could potentially reduce the burden of this serious condition.

**Disclosures:**

**Satchan Takaya, MD FRCPC**, Moderna: Advisor/Consultant

